# Draft genome of *Streptomyces globosus* OSY-MM, a broad-spectrum antimicrobial producer isolated from a cave in South Central Kentucky

**DOI:** 10.1128/mra.00144-26

**Published:** 2026-05-18

**Authors:** Gabriella J. Gephart, Ahmed G. Abdelhamid, Matthew J. Mezydlo, Steven J. Carlson, Zach L. Hawkins, Ahmed E. Yousef

**Affiliations:** 1 Department of Food Science and Technology, The Ohio State University 2647, Columbus, Ohio, USA; 2 Department of Food Science and Human Nutrition, Michigan State University 3078, East Lansing, Michigan, USA; 3 Department of Microbiology, The Ohio State University 2647, Columbus, Ohio, USA; Rochester Institute of Technology, Rochester, New York, USA

**Keywords:** *Streptomyces globosus*, cave isolate, streptothricin, genome, antimicrobial agents

## Abstract

*Streptomyces globosus* OSY-MM was originally isolated from a soil sample collected from a cave in South Central Kentucky and was found to display strong, broad-spectrum antimicrobial activity. *S. globosus* OSY-MM has a 7,277,920 bp genome with 6,476 genes, including biosynthetic gene clusters encoding the production of streptothricin, kirromycin, and venezuelin-like antibiotics.

## ANNOUNCEMENT

*Streptomyces* spp. are well known for producing a large variety of secondary metabolites, including many antimicrobial agents (1). *Streptomyces globosus* OSY-MM was originally isolated from an undisturbed cave passage in South Central Kentucky and was determined to be a prolific antimicrobial producer, as determined by the spot-on-lawn bioassay against *Escherichia coli* K-12 and *Listeria innocua* ATCC 33090 (2). Liquid chromatography-mass spectrometry analysis suggested that *S. globosus* OSY-MM produces the antibiotic streptothricin D. Importantly, streptothricin research is on the rise due to the potential to combat drug-resistant gram-negative bacteria after largely being overlooked due to toxicity concerns (3).

*S. globosus* OSY-MM was grown on tryptic soy agar (BD, Sparks, MD, USA) at 30°C for 96 hours. Resulting spores were then transferred to International *Streptomyces* Project-2 (ISP-2) medium containing 0.4% yeast extract (BD), 1% malt extract (BD), and 0.4% dextrose (Fisher Chemical, Fair Lawn, NJ, USA). The inoculated ISP-2 medium was incubated at 30°C for 48 hours with shaking at 200 rpm. Genomic DNA (gDNA) was extracted from 8 mL of culture using a DNA extraction kit (QIAamp DNA Mini Kit; Qiagen, Germantown, MD, USA) and was cleaned up according to the instructions of the manufacturer. Quantity and quality of gDNA were measured using a spectrophotometer (NanoVue Plus; Biochrom, Holliston, MA, USA). The gDNA was sequenced using a long-read technology (Oxford Nanopore Technologies; Plasmidsaurus, https://www.plasmidsaurus.com). Read statistics (602,875 reads, 1,042,797,863 bases, and coverage of 143×) were provided by Plasmidsaurus. The genome was assembled using Flye 2.9.6 (4). Additionally, short-read sequencing was performed in our laboratory using the Illumina MiSeq platform, as described previously (5). Briefly, libraries were prepared using the Nextera DNA Flex Kit (Illumina, Madison, WI, USA) and were indexed using the Nextera DNA CD Index Kit (Illumina). The libraries were quantified using a fluorimeter (Qubit 4; Invitrogen, Waltham, MA, USA) before sequencing. Paired-end raw reads (2 × 150 bp) were produced. A total of 291,171,146 bp were sequenced, resulting in a coverage of 40×. The Nanopore assembly was polished twice using Racon 1.5.0 (6), and the Nanopore assembly and Illumina reads were compiled utilizing Pilon 1.20 (7). The polished genome was assessed for quality using QUAST 5.3.0 (8) and was determined to contain two contigs (6,695,794 and 582,126 bp), having 74% GC content and an N50 value of 6,695,794 bp. Default parameters were applied for all software used. It was concluded that *S. globosus* OSY-MM has a 7,277,920 bp genome with 6,476 genes and 6,382 coding sequences.

The assembled genome was mined for biosynthetic gene clusters (BGCs) associated with secondary metabolite production utilizing the software antiSMASH 8.0 (9). *S. globosus* OSY-MM contained BGCs encoding the production of streptothricin-, kirromycin-, and venezuelin-like antibiotics. The Genome Taxonomy Database 2.3.2 (10) and Type Strain Genome Server (11) were used for taxonomic classification. In conclusion, *S. globosus* OSY-MM genome encodes novel or rare variants of antimicrobial agents, which may be useful in combating antibiotic-resistant pathogenic bacteria.

## Data Availability

The genome of *Streptomyces globosus* OSY-MM was deposited under GenBank accession number JBTZSZ000000000. The linked BioProject, BioSample, SRA (Illumina), and SRA (Nanopore) accession numbers are PRJNA1414647, SAMN54911780, SRR36994418, and SRR36994417, respectively.
